# Study on the determination method of excitation load in electrical signal simulation for piezoelectric ultrasonic internal inspection of pipelines

**DOI:** 10.1038/s41598-024-84012-z

**Published:** 2024-12-30

**Authors:** Guangli Xu, Jianwen Liu, Qiang Wen, Yuejun Zheng, Liangxue Cai

**Affiliations:** 1 School of Petroleum and Natural Gas Engineering, Southwest Petroleum University, Chengdu, 610500 China; 2 Sichuan Provincial Key Laboratory of Oil and Gas Fire Protection, Chengdu, 610500 China

**Keywords:** Pipeline, Internal inspection, Piezoelectric ultrasonic, Positive piezoelectric, Excitation load, Acoustics, Civil engineering

## Abstract

Accurately determining the initial acoustic field excitation load of a piezoelectric ultrasonic probe is essential for simulating electrical signals and calculating wall thickness during ultrasonic internal inspection of pipelines. A new method for determining the initial excitation load of the acoustic field is proposed, incorporating the focusing effect of the curved surface of pipelines on the ultrasonic signal from the piezoelectric ultrasonic probe. Finite element models were established for the new and old methods using COMSOL software, facilitating the analysis of the initial acoustic field distribution and associated electrical signal characteristics. Scenarios considered included pipelines with and without inner wall defects, and with or without a deviation angle between the pipeline and the probe. The pipeline wall thickness was calculated inversely for each condition. Comparisons with actual wall thickness revealed that the initial excitation load determined by the new method significantly improved accuracy in wall thickness inversion, compared to the published existing method. This indicates that considering the focusing effect of the curved surface of pipelines on ultrasonic signals enhances the accuracy of simulation for piezoelectric ultrasonic internal inspection. This lays the groundwork for developing a digital research and development platform tailored for the ultrasonic internal detectors of pipeline.

## Introduction

Pipeline systems are vital to the oil, gas, and chemical industries, with their safety and integrity influencing operational efficiency and environmental protection. As pipelines age, the risk of defects such as corrosion and cracks increase, necessitating regular inspection^1^. Among various non-destructive testing technologies, piezoelectric ultrasonic testing is notable for its safety, efficiency, high precision, and long-distance detection capabilities^2,3^.

**Fig. 1:**
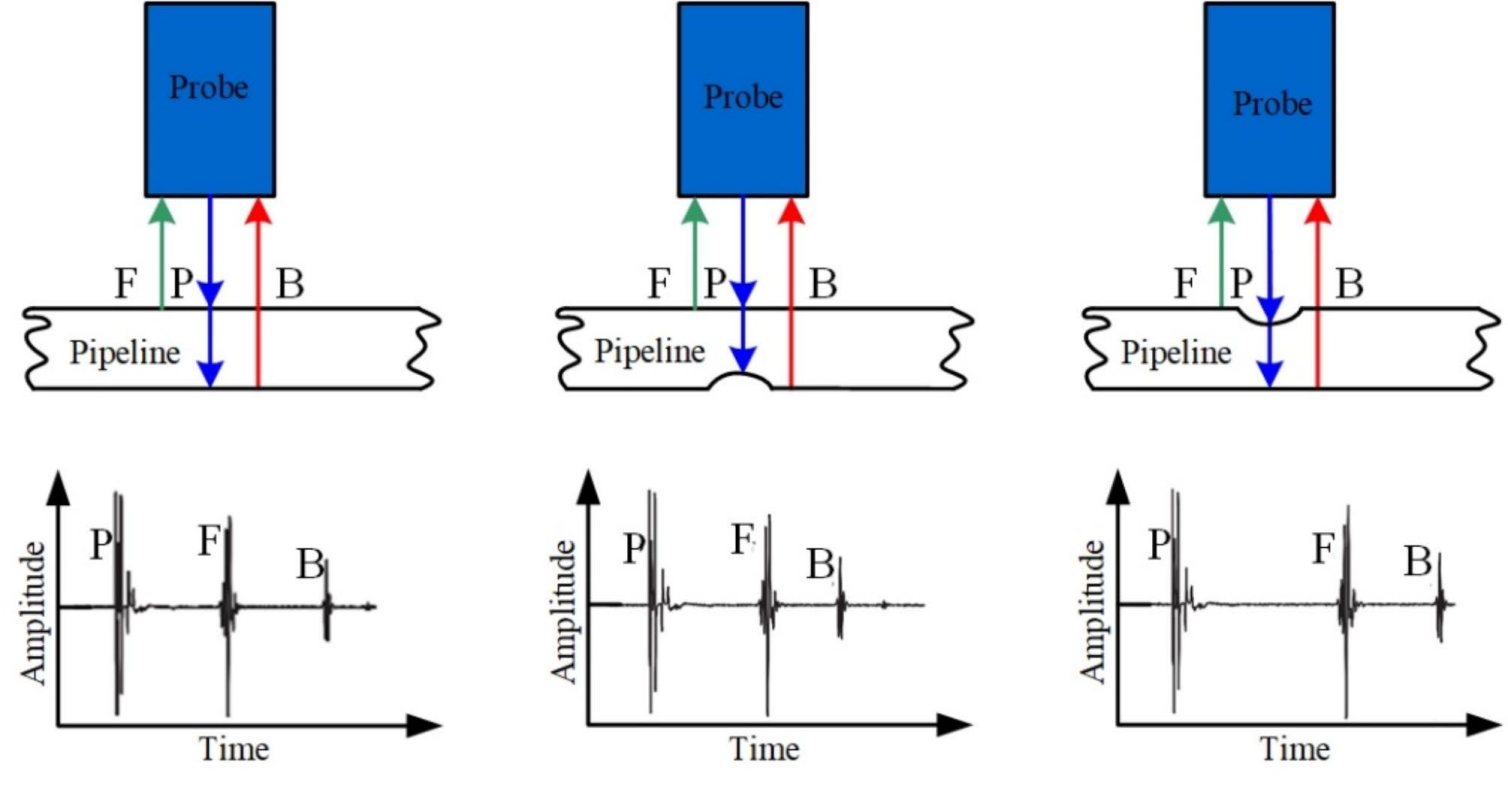
Ultrasonic internal inspection Principle for pipeline defects. P: pulse signal emitted by the probe; F: pulse echo from the internal wall of the pipeline; B: pulse echo from the external wall of the pipeline.

When subjected to specific excitation loads, the ultrasonic probe emits ultrasonic waves, which then propagate both between the probe and the pipeline and within the pipeline itself, as illustrated in Fig. 1. The reflected ultrasonic waves are then received by the probe. Through the analysis of pulse-echo signals, details such as pipeline wall thickness, defects can be acquired. Researchers worldwide have conducted extensive studies on ultrasonic inspection of pipeline defects, using two main approaches: laboratory experiments and numerical simulations^4,5^.

**Fig. 2:**
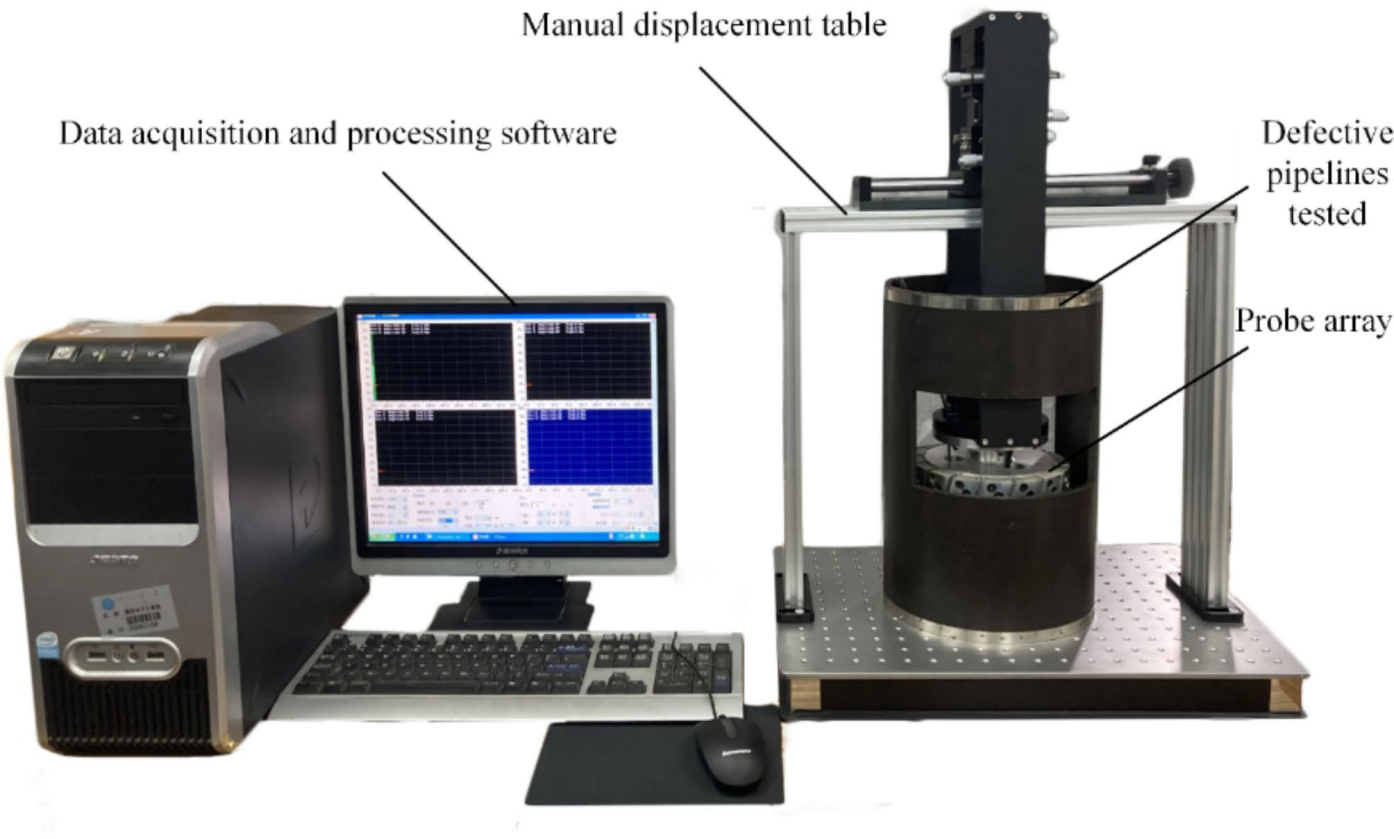
Experimental equipment for ultrasonic detection of a pipeline.

In laboratory experiments, an experimental platform is commonly utilized to carry out tests. The experimental equipment for piezoelectric ultrasonic internal inspection of pipelines is shown in Fig. 2, which mainly includes a test pipeline segment with defects, a probe array chamber, piezoelectric ultrasonic probes, a manual displacement table, a multi-channel ultrasonic emission card, and a data acquisition and processing system^6^. Among them, the piezoelectric ultrasonic probes serve as the core sensing elements, generating and receiving ultrasonic waves to detect pipeline defects with high precision; the manual displacement table enables precise adjustment of the spatial orientation between the ultrasonic probe and the pipeline, reproducing the actual state during online pipeline inspection; the data acquisition and processing system ensures accurate and reliable performance by facilitating ultrasonic signal acquisition, noise reduction, and signal amplification.

After acquiring ultrasonic echo signals under various conditions, the ultrasonic signals are processed and analyzed employing different signal processing techniques, such as amplitude reduction^6^, wavelet transform^7^, fast Fourier transform^8^, 1.5-dimensional spectrum^9^, and overlapped echo separation with matching pursuit^10^. These methods facilitate the establishment of quantitative relationships between pipeline dimensional parameters (such as wall thickness and defects) and echo signal characteristics. These studies have established a foundation for the development of ultrasonic internal detectors of pipeline. However, slight fluctuations in environmental parameters during experiments can result in significant variations in the echo signal, and the scarcity of measurable parameters compromises the accuracy of quantifying the echo signal to some extent. Numerical simulation techniques offer a crucial complement to address this limitation.

Esmaeil Mirmahdi et al.^11^ employed a Gaussian pulse function as their simulated excitation signal, and used COMSOL software to perform a three-dimensional (3D) numerical simulation of the defect detection using ultrasonic phased array probes, which were directly attached to the outer surface of the stainless-steel pipeline. The simulation results were in good agreement with the measured results, which fully confirms the effectiveness of COMSOL software in simulating the ultrasonic detection of pipeline defects^12,13^.

Unlike ultrasonic phased array detection, when the detector moves through the liquid-filled pipeline at a specific velocity, there exists a defined lift height between the ultrasonic probe and the pipeline. As illustrated in Fig. 1, the piezoelectric ultrasonic testing process encompasses three stages: signal emission, propagation, and reception. When subjected to an electrical excitation, the piezoelectric ultrasonic probe generates ultrasonic pulse signals through its inverse piezoelectric effect. After propagating through space, these reflected ultrasonic pulse echoes return to the probe, where they are converted into electrical signals through the positive piezoelectric effect^14^. The critical information about the pipeline including wall thickness, as well as the size and location of defects, is embedded in these electrical signals^15–17^. Consequently, detailed insights into pipeline defects can be deduced by processing and analyzing electrical signals. When employing numerical simulation methods, a 3D finite element model should be established incorporating the probe, liquid medium, and pipeline. According to the COMSOL software manual, the computational requirements for creating a 3D finite element analysis model encompassing the entire process of signal emission, propagation, and reception exceed 1 TB of memory. As a result, the propagation process of ultrasonic waves between the probe and the pipeline can be simplified to a two-dimensional (2D) model. Xu Guangli et al.^18^ employed COMSOL software to develop a physical model of the piezoelectric ultrasonic probe and simulate its operating state within a pipeline. This model encompasses the entire detection process, from the excitation of piezoelectric ultrasonic wave, its propagation through the probe and the pipeline, to its conversion into electrical signals. However, the focusing effect of the curved surface of pipeline on ultrasonic signals was overlooked when converting the 2D acoustic pressure simulation results obtained at the probe surface into a 3D initial excitation load for simulating the positive piezoelectric effect of the probe. This omission led to an insufficiently accurate 3D initial excitation load for the positive piezoelectric process. Therefore, optimizing this initial excitation load can further enhance detection accuracy and precision^19,20^.

To account for the focusing effect of the curved pipeline surface on ultrasonic signals generated from a piezoelectric probe, a novel method is introduced for determining the initial excitation load of acoustic field in the simulation of electrical signals for ultrasonic internal inspection of pipelines. Finite element models were established for both the new and published old methods by using COMSOL software to compare and analyze the initial acoustic field distribution, acoustic power and corresponding electrical signal characteristics under various conditions. Scenarios considered included pipelines with and without inner wall defects, and with or without a deviation angle between the pipeline and the ultrasonic probe. By comparing simulated wall thickness with actual measurements, the new method has confirmed to improve detection accuracy. This advancement facilitates the establishment of a digital research and development platform for ultrasonic internal inspection of pipelines.

## Determination of excitation load

### Establishment of ultrasonic internal inspection model

**Fig. 3:**
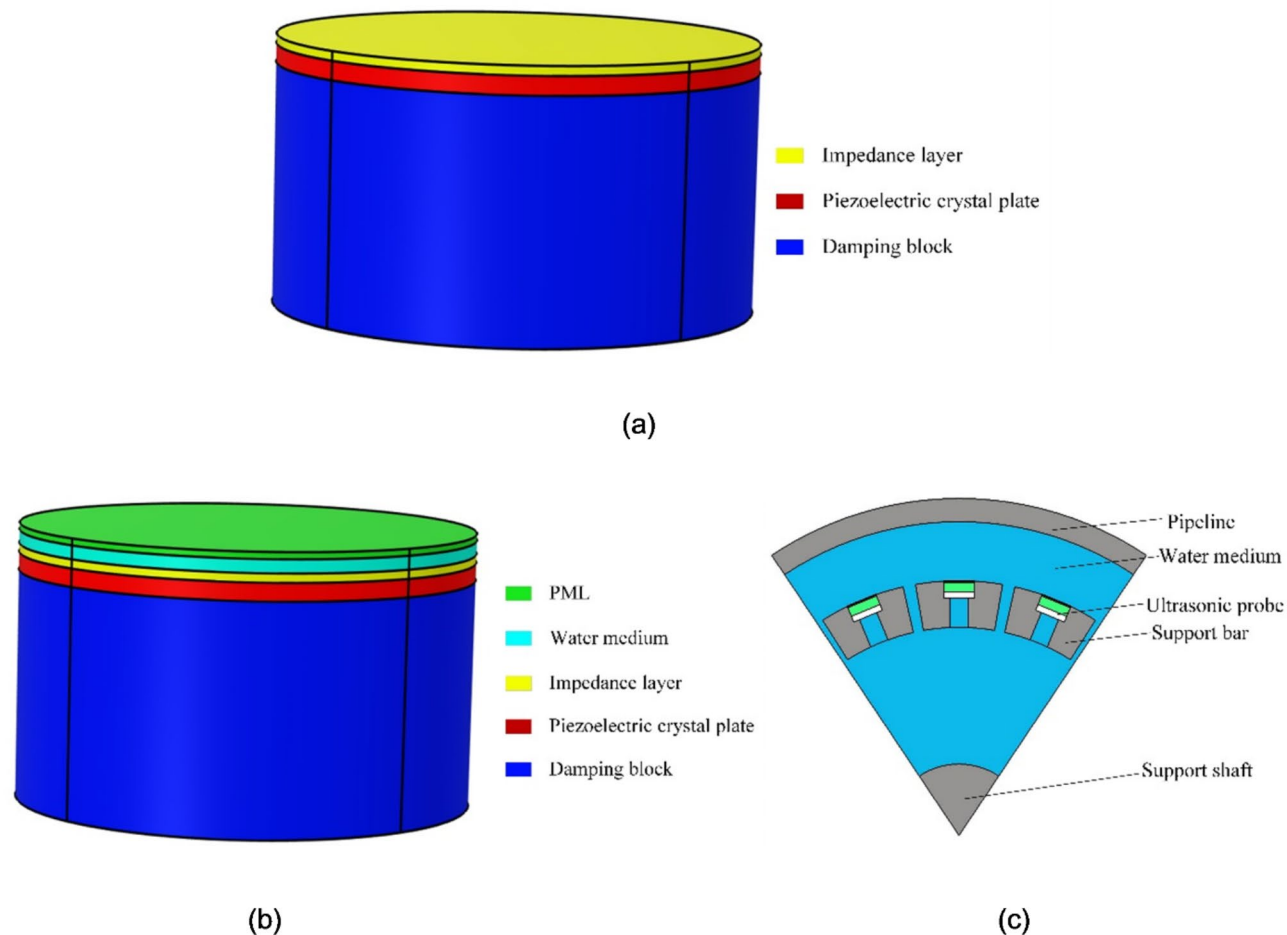
Physical models for simulating ultrasonic internal inspection of pipeline: (**a**) positive piezoelectric process model; (**b**) inverse piezoelectric process model; (**c**) ultrasonic wave propagation model.

Using the parameters listed in Table 1 and the simulation strategy developed in our prior study (Reference 18), the physical model was intentionally reused and extended to ensure consistency and comparability^18^. This approach facilitates a direct evaluation of the novel excitation load determination method introduced in this study under conditions similar to those previously explored. In the simulations, both the inverse and positive piezoelectric processes were modeled in three dimensions. For the positive piezoelectric process as shown in Fig. 3a, the model consisted of a cylindrical structure with a diameter of 10 mm, composed of a damping layer, a piezoelectric crystal plate, and an impedance layer. In the inverse piezoelectric process as shown in Fig. 3b, a water medium and a Perfectly Matched Layer (PML) were also included in the model. Among them, the PML intercepts the water medium and absorbs the propagating ultrasonic waves in the fluid, effectively reducing boundary reflections. The pipe under inspection was a seamless Q235 steel pipe with a diameter of 219 mm and a wall thickness of 8.0 mm. During inspection of pipelines, ultrasonic waves propagate between the probe and the pipeline. Given the complexity of the acoustic wave propagation, and the time-consuming nature of 3D calculations, a 2D numerical simulation is advisable^18^. Therefore, the propagation of ultrasonic waves between the probe and the pipeline, as shown in Fig. 3c, was simulated in two dimensions in this study.

**Table 1:** Ultrasonic probe material properties and basic parameters.

Component	Material	Density/kg/m^3^	Height/mm	Pressure wave velocity/m/s	Shear wave velocity/m/s
Impedance layer	Corundum flakes	3900	0.195	6310	3080
Piezoelectric crystal plate	PZT-5 H	7500	0.390	3900	2850
Damping block	Epoxy resin with tungsten powder	1180	5.000	3200	1600

Under ideal conditions (a defect-free pipeline without deviation angle between the pipeline and the probe), COMSOL software was used to perform 3D simulations of the behavior of the PZT-5 H piezoelectric probe (center frequency of 5 MHz) in both inverse and direct piezoelectric processes, with distilled water serving as the coupling agent. Two and a half cycles of square wave pulse were used as the excitation load for the inverse piezoelectric process 3D simulation, resulting in 3D acoustic waves on the probe surface. Then, the 2D acoustic wave along the probe diameter direction was extracted as the initial load for the acoustic wave propagation simulation, resulting in a 2D acoustic signal that returns to the probe surface through the pipeline. These 2D simulation results were then converted into 3D acoustic signals, serving as the excitation load for the 3D numerical simulation of the positive piezoelectric process.

**Fig. 4:**
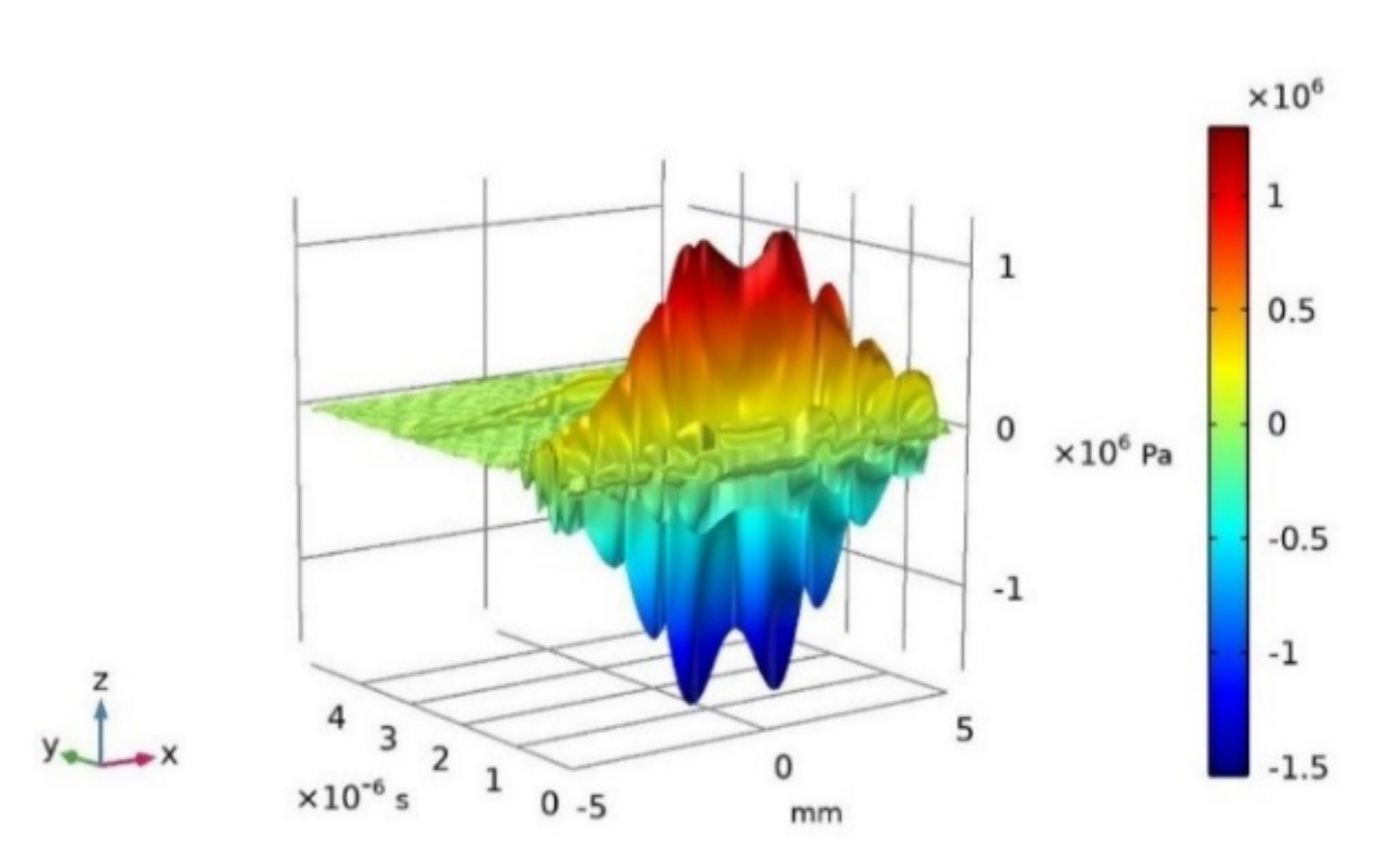
2D ultrasonic echo acoustic field diagram under ideal condition of no defect on pipeline and no deviation angle between pipeline and probe.

The propagation speed of ultrasonic wave in water^6^ is 1468 m/s, and the lift-off height, i.e., the vertical distance from the center of the probe to the inner wall of pipeline, is 19 mm. Hence, the theoretical time for the ultrasonic echo to reach the probe surface is equal to twice the lift-off height divided by the propagation speed of ultrasonic wave in water, specifically 25.88 µs (= 2 × 19/1468 mm/(m/s)). The simulated 2D ultrasonic echo acoustic field with a time range of 25.6 ~ 30.6 µs along the diameter of the ultrasonic probe (10 mm) under ideal condition is depicted in Fig. 4. In which, red indicates wave peaks, blue indicates wave valleys, and sound pressure amplitude undergoes drastic changes over time. The peak sound pressure is associated with the surface echo. Over time, a subsequent increase in sound pressure corresponds to a primary bottom echo reflected from the outer surface of the pipeline. However, the 2D sound pressure distribution shown in Fig. 4 cannot be directly applied as the excitation load for the positive piezoelectric process of the 3D ultrasonic probe depicted in Fig. 3c. Consequently, it is necessary to convert the acoustic field from 2D x-coordinate, t-time, and sound pressure into a 3D *x* and *y* coordinates, t-time, and sound pressure.

### Centre-symmetric conversion method

Reference^18^ deposits that the ultrasonic echo acoustic field is symmetric about the midpoint of the piezoelectric crystal plate, and proposes a centre-symmetric conversion method. In which, the data along the radius of the impedance layer of the piezoelectric crystal plate as shown in Fig. 4 are copied through rotating around the midpoint of the probe at 5° intervals. This 3D acoustic field, obtained by rotational replication of sound pressure data along the radius, is exerted on the upper surface of the impedance layer as a boundary load pressure, serving as the initial excitation load for simulating the positive piezoelectric process. This approach is called as the old conversion method.

### Conversion method considering focusing effect of curved surface of pipeline on ultrasonic waves

**Fig. 5:**
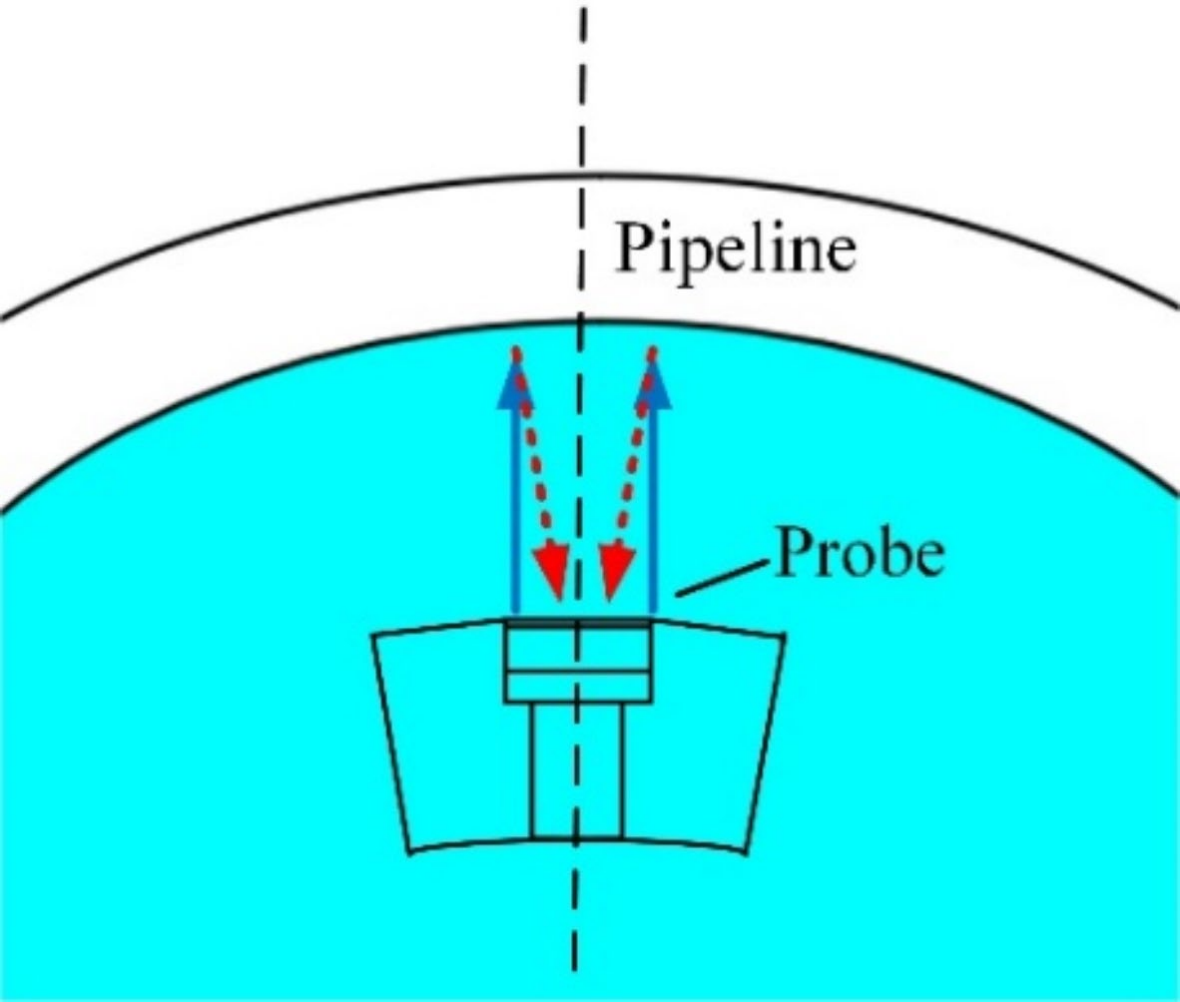
The focusing effect of the pipeline wall on ultrasonic signals.

If the inner wall surface of a pipeline is equivalent to a flat plate, the acoustic field returning to the probe through the pipeline wall should be centrosymmetric. This means that the sound pressure at positions equidistant from the circle’s center is a same value. Should this be the case, centre-symmetric conversion method is indeed correct. However, the inner wall surface of a pipeline is actually curved, leading to a focusing effect on the ultrasonic signal, as illustrated in Fig. 5. Additionally, due to the curvature of the pipeline wall, the acoustic waves emitted by the probe reach the inner wall surface over a period of time rather than at the same instant. Consequently, the published centre-symmetric conversion method has certain errors compared to actual conditions.

**Fig. 6:**
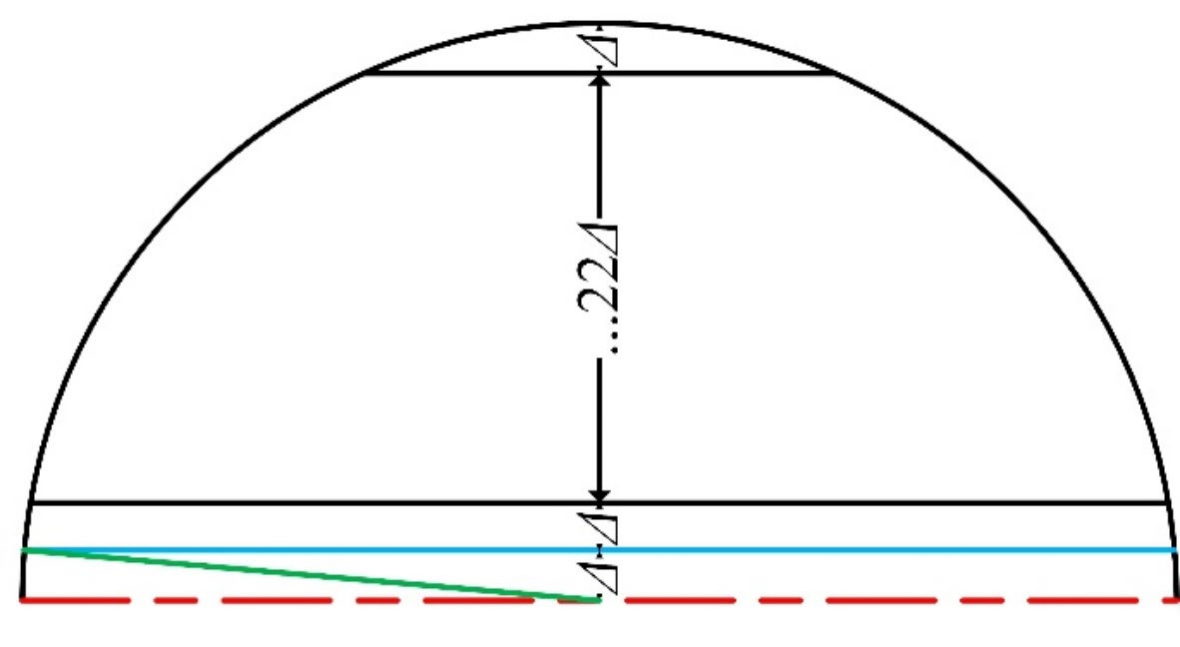
Schematic diagram of a new conversion method for the excitation load in 3D simulation of positive piezoelectric process.

The echo acoustic field on the probe is symmetrical about its diameter in 2D sound pressure propagation simulation. This symmetry implies that the 3D acoustic field on the probe can be reconstructed through acquiring the sound filed on half of the probe and applying axisymmetry. To leverage this, the length of piezoelectric crystal plate in the 2D sound pressure propagation model was gradually reduced from 10 mm to 0 with an interval (*Δ*) of 0.2 mm, as illustrated in Fig. 6. This approach obtains the 2D acoustic field for various radius of piezoelectric crystal plate after the inverse piezoelectric process and wave propagation. The acoustic field data corresponding to the time interval of 25.6 ~ 30.6 µs were collected for each radius of the piezoelectric crystal plate. This time range was determined based on the ultrasonic wave propagation characteristics under current conditions. It would need adjustment if the lift-off height or coupling agent changes. By combining these data, the acoustic field within the semicircular region was reconstructed. This semicircular acoustic field was then symmetrically replicated to generate the 3D initial acoustic field load required for simulating the positive piezoelectric process. This approach is called as the optimized conversion method.

## Comparison of two excitation load conversion methods under ideal condition

### Acoustic field distribution

**Fig. 7:**
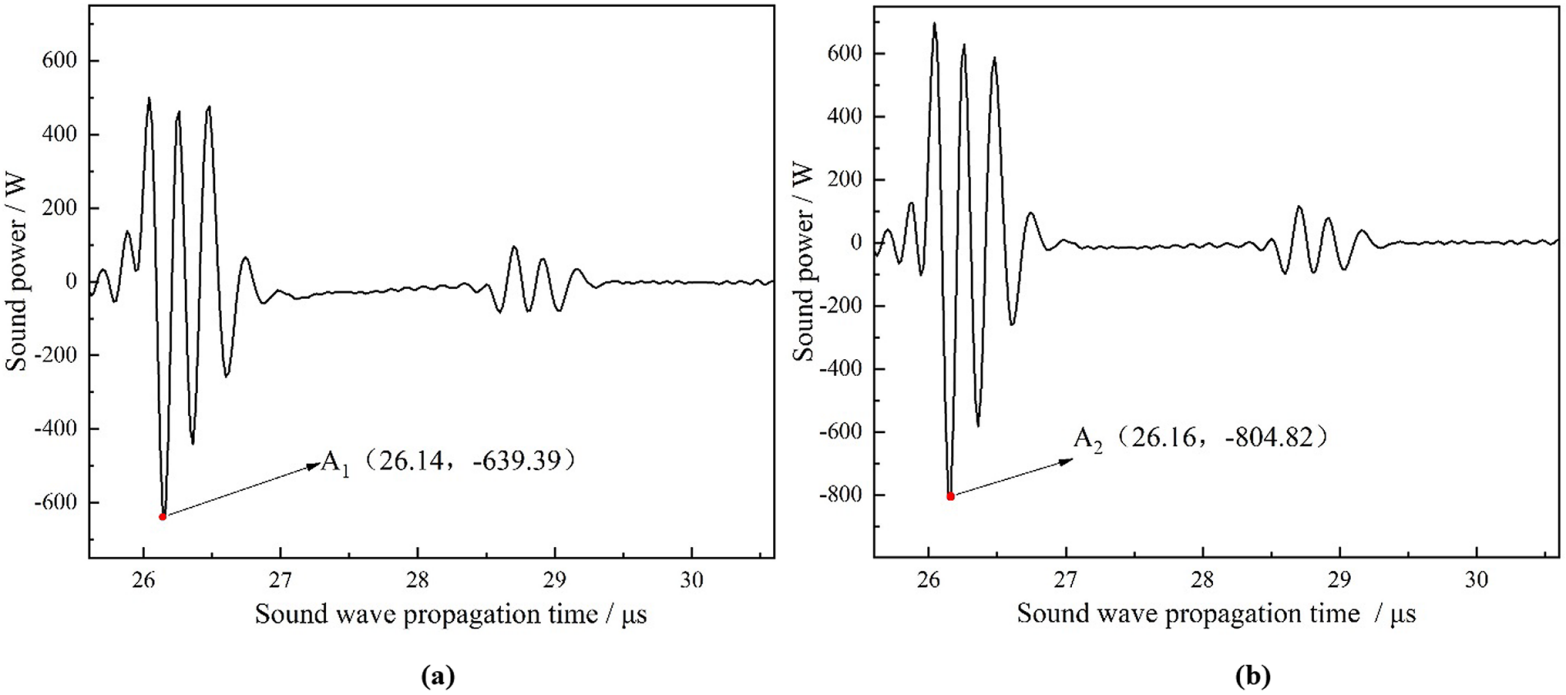
Average instantaneous sound power of two conversion methods under ideal condition: (**a**) centre-symmetric conversion method; (**b**) optimized conversion method.

Sound power represents the total energy emitted by a sound source per unit time. The average instantaneous sound power of the 3D acoustic field, derived from the two conversion methods, is depicted in Fig. 7. Notably, there are differences in the amplitude of the maximum trough point and its corresponding time (surface echo position) between the two methods. This discrepancy indicates the need for further analysis of the 3D acoustic field around 26.16 µs.

**Fig. 8:**
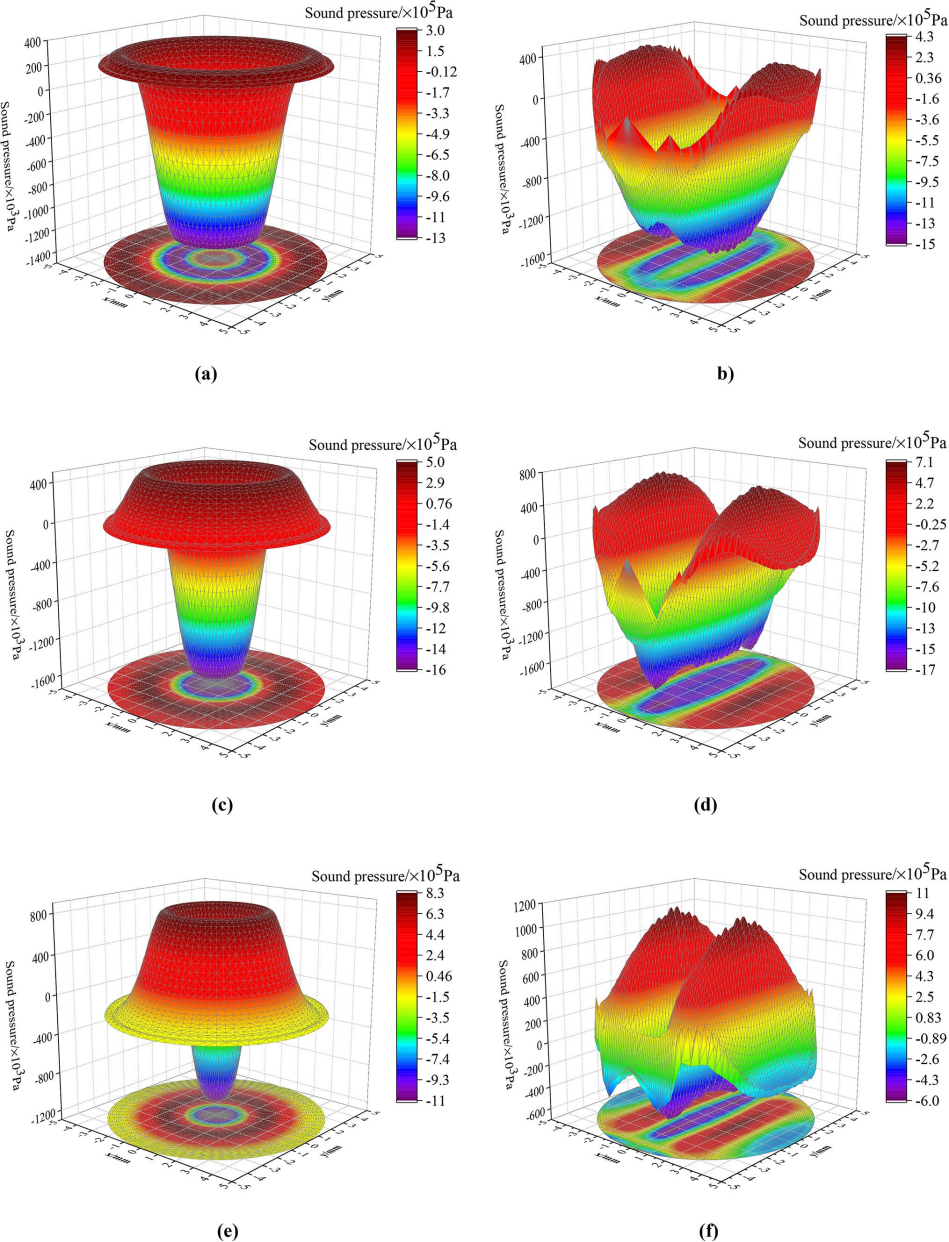
3D initial acoustic field of the two methods at different times under ideal condition. (**a**) *t* = 26.12 µs - old method; (**b**) *t* = 26.12 µs - optimized method; (**c**) *t* = 26.16 µs - old method; (**d**) *t* = 26.16 µs - optimized method; (**e**) *t* = 26.20 µs - old method; (**f**) *t* = 26.20 µs - optimized method.

The 3D initial acoustic field obtained using the two conversion methods exhibit significant differences, as shown in Fig. 8. Owing to the highly idealized assumptions of the old conversion method, all the sound pressure amplitude of positions with a same distance from the center of the probe are equal, resulting in a symmetrical 3D acoustic field around the central axis. In contrast, the 3D acoustic field, derived from the optimized conversion method, exhibits symmetry about the *Z-X* plane passing through the origin. This observation indicates that the 3D acoustic field is more accurate when accounting for the focusing effect of curved pipe surface on the ultrasonic signal. Further analysis of Fig. 8b,d,f shows that at 26.12 µs, the negative pressure at the wave valley is significantly greater than the positive pressure at the wave peak, thereby negative sound pressure is dominant. At 26.16 µs, the sound pressure at the valley continues to decrease, while that at the peak increases, with negative pressure remains dominant. At 26.20 µs, the negative sound pressure at the valley begins to decrease, and its acting region reduces; while the positive sound pressure at the peak increases, and its acting region expands. Summarily, in the time range from 26.12 µs and 26.16 µs, both negative and positive pressures on the piezoelectric crystal plate gradually intensifies, with the negative pressure surging more than the positive, thereby making the negative pressure predominant. From 26.16 µs to 26.20 µs, the negative pressure gradually diminishes while the positive pressure continues to rise, leading to a transition where the negative pressure undergoes from being dominant to gradually equalizing with the positive pressure. From the perspective of force acting on a piezoelectric crystal plate, the probe experiences a continuously increasing downward force from 26.12 µs to 26.16 µs, reaching its maximum at 26.16 µs. Following this, from 26.16 µs to 26.20 µs, the downward force weakens while the upward force gradually increases.

### Electrical signal

**Fig. 9:**
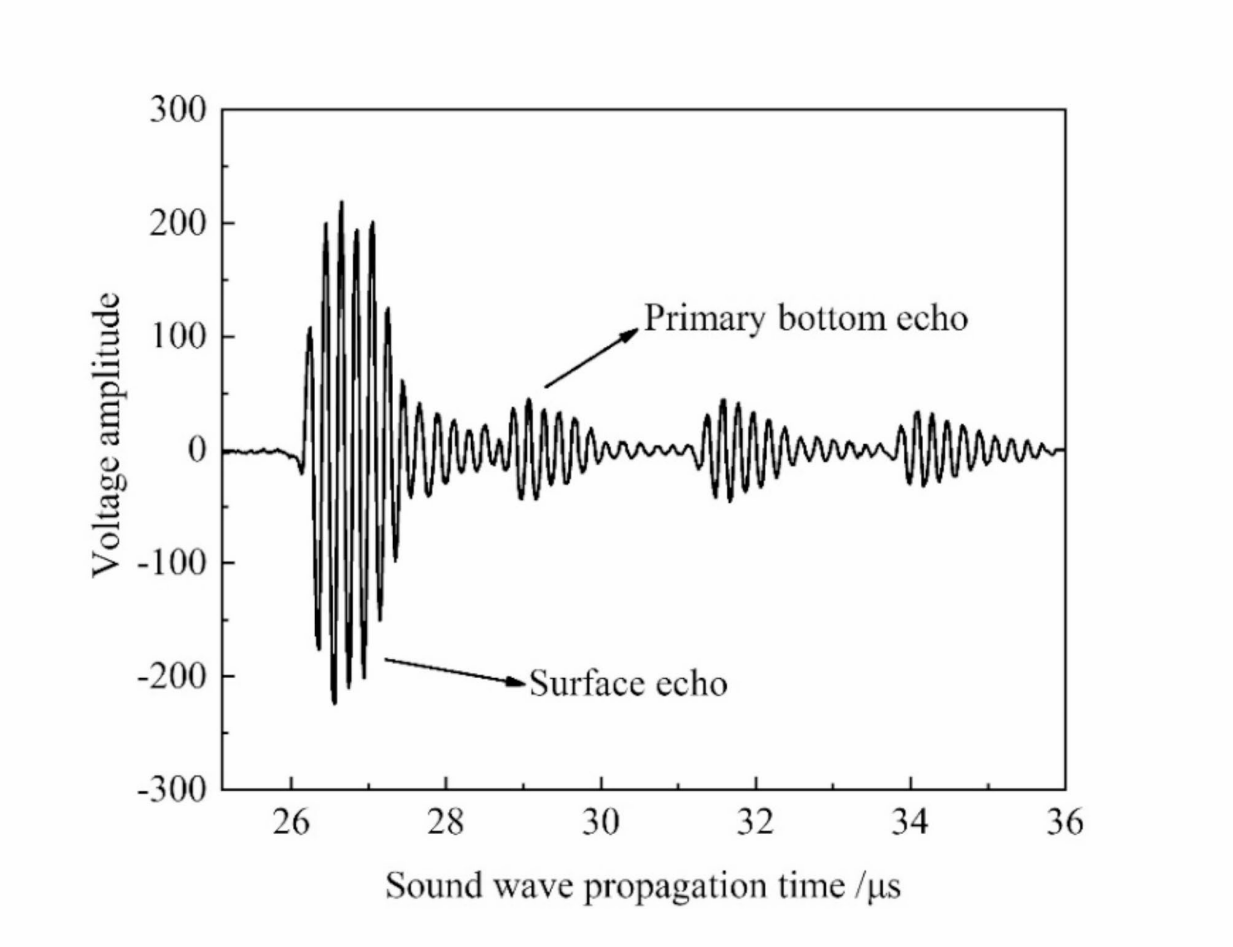
The measured electrical signal.

The signal under ideal condition, acquired using the same experimental equipment and procedures as described in the literature^18^, is shown in Fig. 9. When ultrasonic wave propagates through water to the inner wall of the pipeline, the significant difference in acoustic impedance between water and steel causes most of the reflected energy to be initially received by the probe as the surface echo. A smaller portion of the energy refracts into the pipeline wall, then propagates to the outer wall before being reflected back to the inner wall. Additionally, part of this energy refracts into the water, while the remaining portion is reflected back to the outer wall. This process is repeated until the energy is dissipated, resulting in multiple bottom echoes due to the repeated refractions and reflections between the inner and outer walls of the pipeline, as illustrated in Fig. 9. The maximum absolute values of the voltage amplitudes for both the surface and the primary bottom echoes in Fig. 9 were identified as feature points. The ratio between these two values, 4.98 (calculated as 224/45), was then compared with the simulation results.

**Fig. 10:**
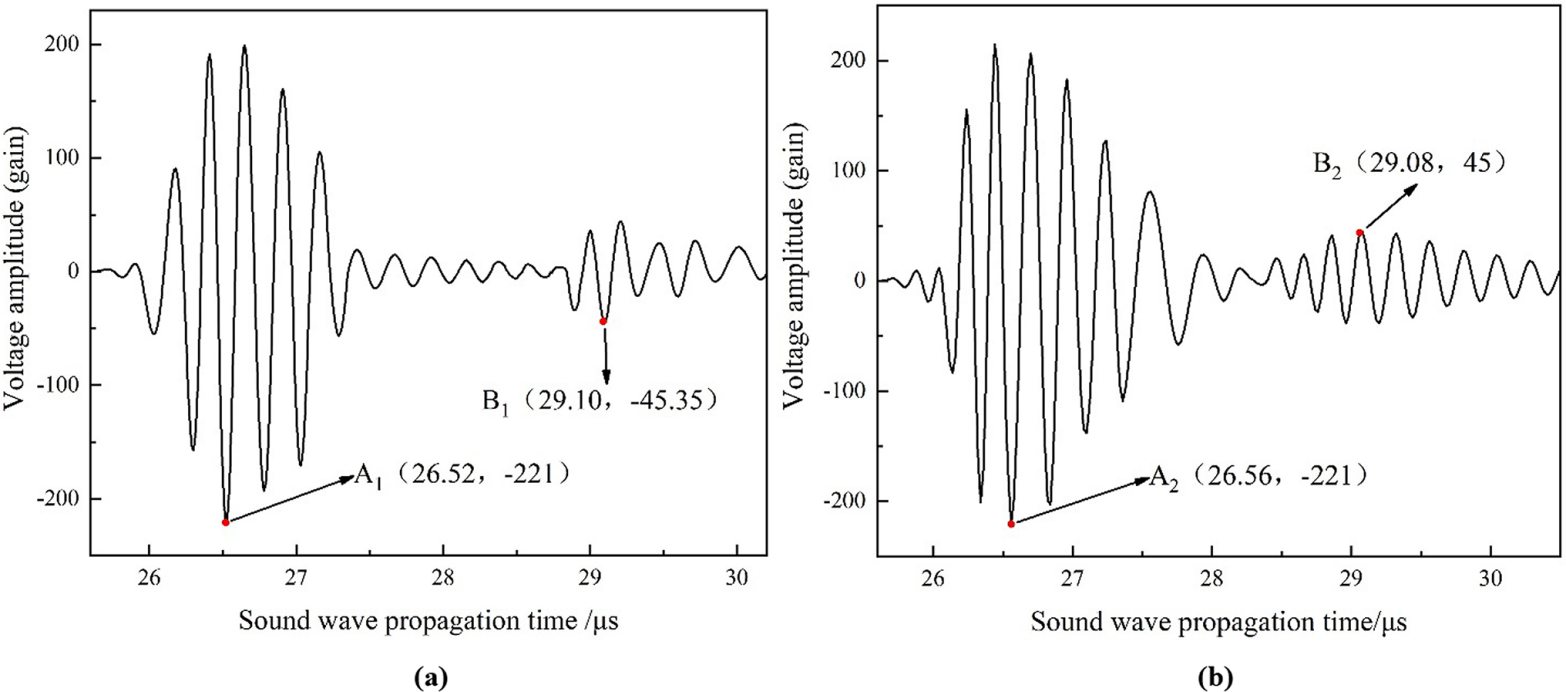
The simulated electrical signal diagram of two conversion methods under ideal condition: (**a**) centre-symmetric conversion method; (**b**) optimized conversion method.

The 3D acoustic field obtained from the two conversion methods were used as initial excitation loads to simulate the positive piezoelectric process of a piezoelectric crystal plate. The approach enabled the determination of how voltage amplitude varies over the course of acoustic wave propagation, as depicted in Fig. 10. In Fig. 10a, the maximum absolute value of the surface echo obtained through the centre-symmetric conversion method, referred to as point A1, is observed at (26.52 µs, − 221 V). The maximum absolute value of the primary bottom echo, designated as point B1, occurs at (29.10 µs, − 45.35 V). The amplitude ratio of these absolute values is calculated to be 4.87 (equivalent to 221/45.35), with a deviation of − 2.21% from the measured value of 4.98. By utilizing the propagation speed of acoustic wave in the impedance layer (6000 m/s) and considering the thickness of the impedance layer (0.195 mm), the propagation time of the echo through the impedance layer is calculated to be 0.0325 µs. Based on the time when the piezoelectric probe receives the surface echo (26.52 µs), the propagation time of the echo through the impedance layer (0.0325 µs), and the propagation speed of the acoustic wave in water (1468 m/s), the lift-off height from the probe to the inner wall surface of the pipeline can be calculated as 19.418 mm, with an error of + 2.20% from the actual value of 19.0 mm. Furthermore, based on the time difference between the surface echo and the primary bottom echo, and the propagation speed of the acoustic wave in steel (6000 m/s), the simulated wall thickness is calculated to be 7.74 mm, with an error of + 3.47% from the actual wall thickness of 7.48 mm. In Fig. 10b, the maximum absolute value of the surface echo obtained through the optimized conversion method, denoted as point A2, is observed at (26.56 µs, -221 V). The maximum absolute value of the primary bottom echo, represented as point B2, occurs at (29.08 µs, -45 V). The amplitude ratio of these absolute values is calculated to be 4.91 (equivalent to 221/45), with a deviation of -1.41% from the measured value of 4.98. Using the same algorithm, the lift-off height, the pipeline wall thickness, and their corresponding errors can be calculated, as shown in Table 2. The results indicate that the accuracy of wall thickness simulated using the optimized initial acoustic field is improved by 2.40% compared to the old method.

**Table 2:** Comparison of simulation results obtained by two methods and the measurements under ideal condition.

Items	Feature point ratio	Relative error/%	Lift-off height/mm	Relative error/%	Wall thickness/mm	Relative error/%
Measurement	4.98	/	19.00	/	7.48	/
Simulation by the old method	4.87	−2.21	19.418	+ 2.20	7.74	+ 3.47
Simulation by the optimized method	4.91	−1.41	19.447	+ 2.35	7.56	+ 1.07

### Comparison of two excitation load conversion methods under non-ideal conditions

As previously mentioned in Sect. 1, the ideal condition is defined as a defect-free pipeline without deviation angle between the pipeline and the probe. However, over time since a pipeline was commissioned, defects may arise on both the inner and outer walls due to corrosion and other reasons. Furthermore, the deviation angle between the ultrasonic probe’s wave emission direction and the radial line of the pipeline (a line passing through the geometric center of the defect on the pipeline’s inner wall) is defined as the deviation angle between the pipeline and the ultrasonic probe^6^. During the operation of the ultrasonic internal inspection system, several factors contribute to this deviation angle. These include the geometry of the surface under inspection, the piezoelectric crystal plate fabrication, the motion control method of the probe, the spatial constraints of the inspection environment, and the ovality of the pipeline. All these conditions are classified as non-ideal conditions.

### Electrical signal of a defective pipeline without deviation angle

**Fig. 11:**
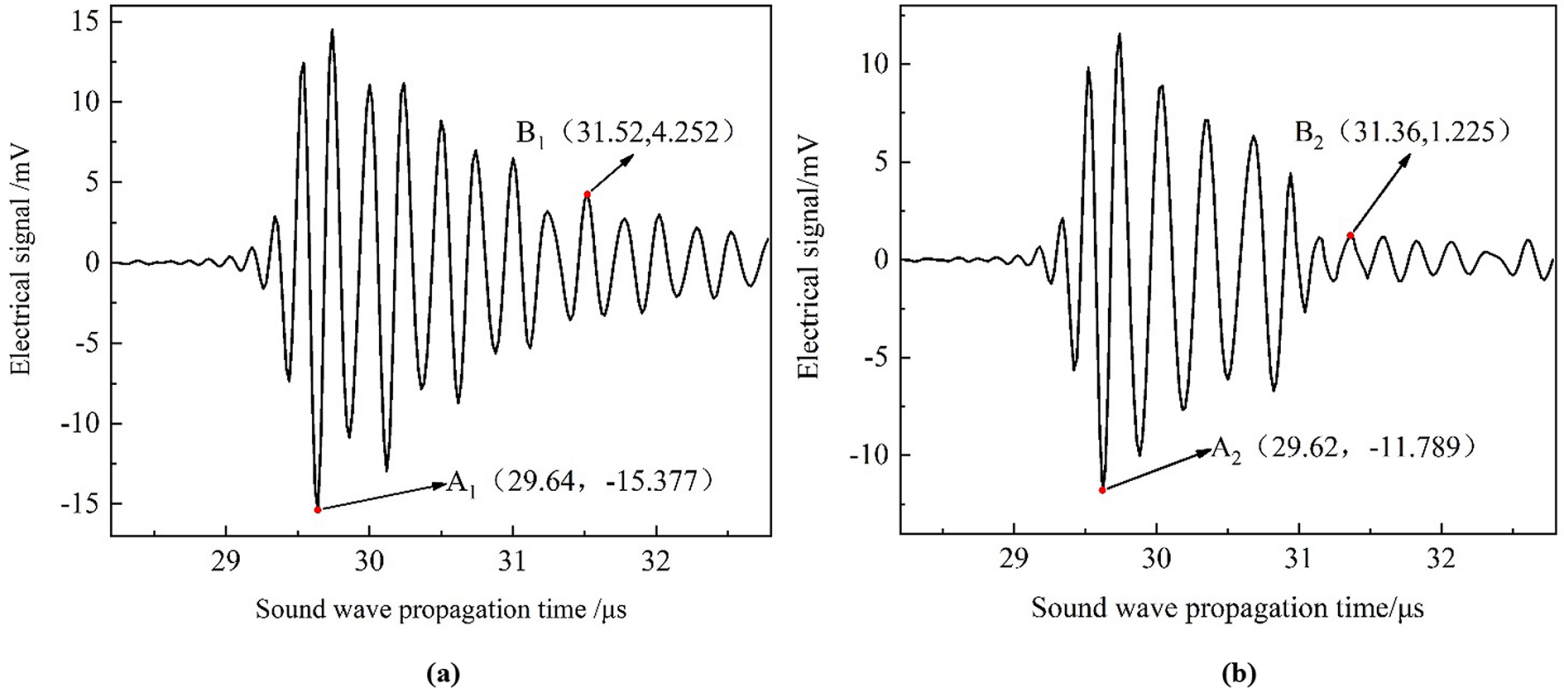
The simulated electrical signal diagram of two conversion methods when the probe directly faced the pipeline defect: (**a**) centre-symmetric conversion method; (**b**) optimized conversion method.

Utilizing the finite element analysis model of the PZT positive piezoelectric process, the electrical signal was analyzed when the probe directly faced the pipeline defect. The defect is positioned on the inner wall of the pipeline with a depth of 2.4 mm, and dimensions of 10 mm in both length and width, to verify the accuracy of the optimized conversion method in the presence of pipeline defects. Theoretical calculations indicate that the ultrasonic pulse takes 29.15 µs to travel from the probe to the inner wall of the defective pipeline, reflect off the inner wall, and return to the probe. Therefore, the 2D acoustic field signals in the time range of 28.2 to 33.2 µs were extracted. These signals were then used to generate the initial excitation load for the simulation of the positive piezoelectric process using both conversion methods. This approach enabled the determination of how voltage amplitude varies over propagation time, as shown in Fig. 11. Corresponding feature points were extracted from the results. In Fig. 11a, the maximum absolute value of the surface echo obtained through the centre-symmetric conversion method, referred to as point A1, is observed at (29.64 µs, − 15.377 V). The maximum absolute value of the primary bottom echo, designated as point B1, occurs at (31.52 µs, + 4.252 V). While in Fig. 11b, the maximum absolute value of the surface echo obtained through the optimized conversion method, denoted as point A2, is observed at (29.62 µs, − 11.789 V). The maximum absolute value of the primary bottom echo, represented as point B2, occurs at (31.36 µs, + 1.225 V). Following the same calculation approach as described in “Centre-symmetric conversion method” Section, the lift-off height from the probe to the inner wall of the pipeline and the wall thickness were determined for both conversion methods, as shown in Table 3. The optimized conversion method was found to result in an 8.26% improvement in the accuracy of the calculated wall thickness compared to the old method.

**Table 3:** Comparison of simulation results obtained by two methods and the measurements when the probe directly faced the pipeline defect.

Items	Lift-off height/mm	Relative error/%	Wall thickness/mm	Relative error/%
Measurement	21.40	/	5.08	/
Simulation by the old method	21.708	+ 1.44	5.64	+ 11.02
Simulation by the optimized method	21.693	+ 1.37	5.22	+ 2.76

### Electrical signals of a defective pipeline with circumferential deviation angle

**Fig. 12:**
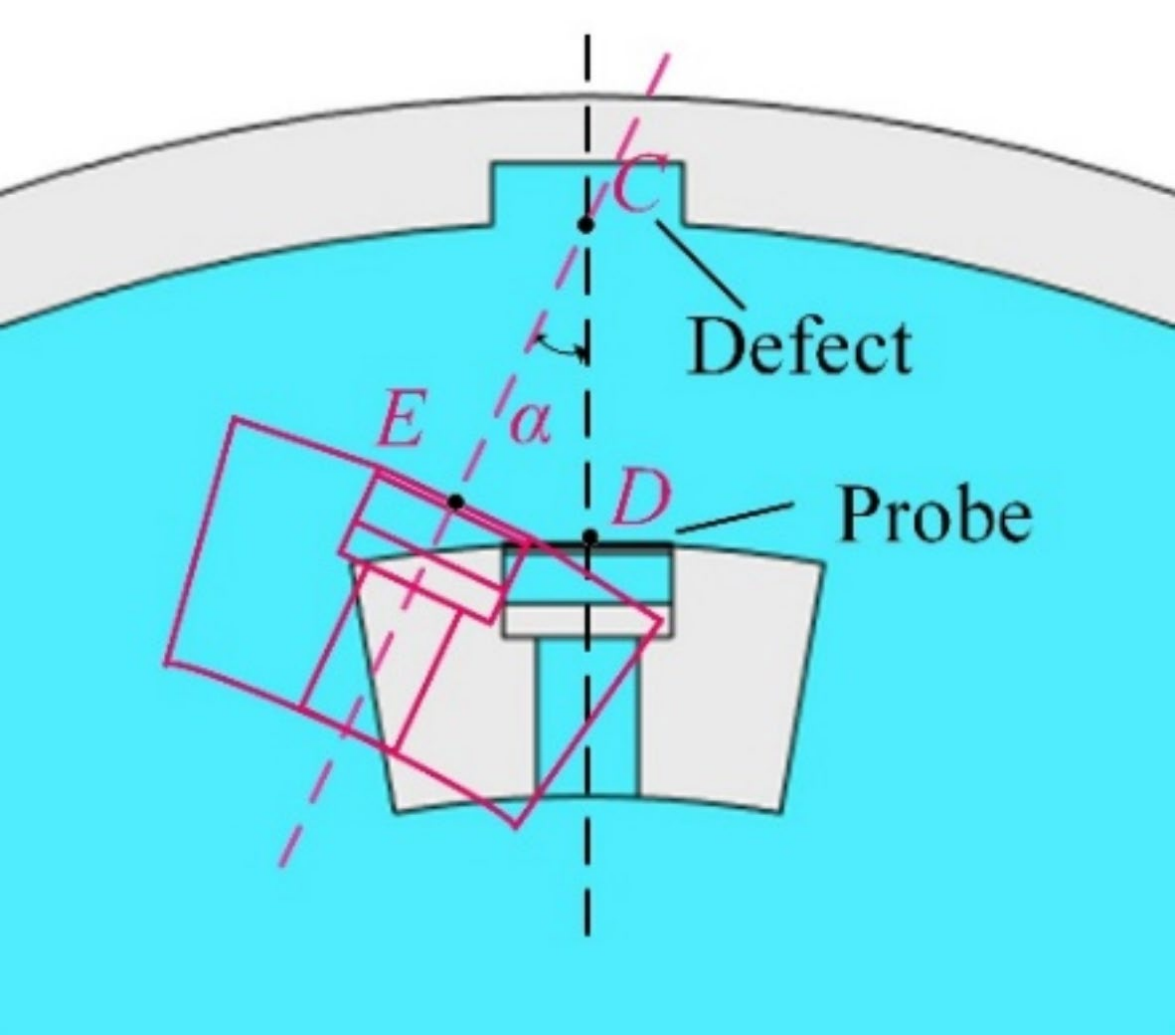
Schematic diagram of circumferential deviation angle between the probe and the defective pipeline.

**Fig. 13:**
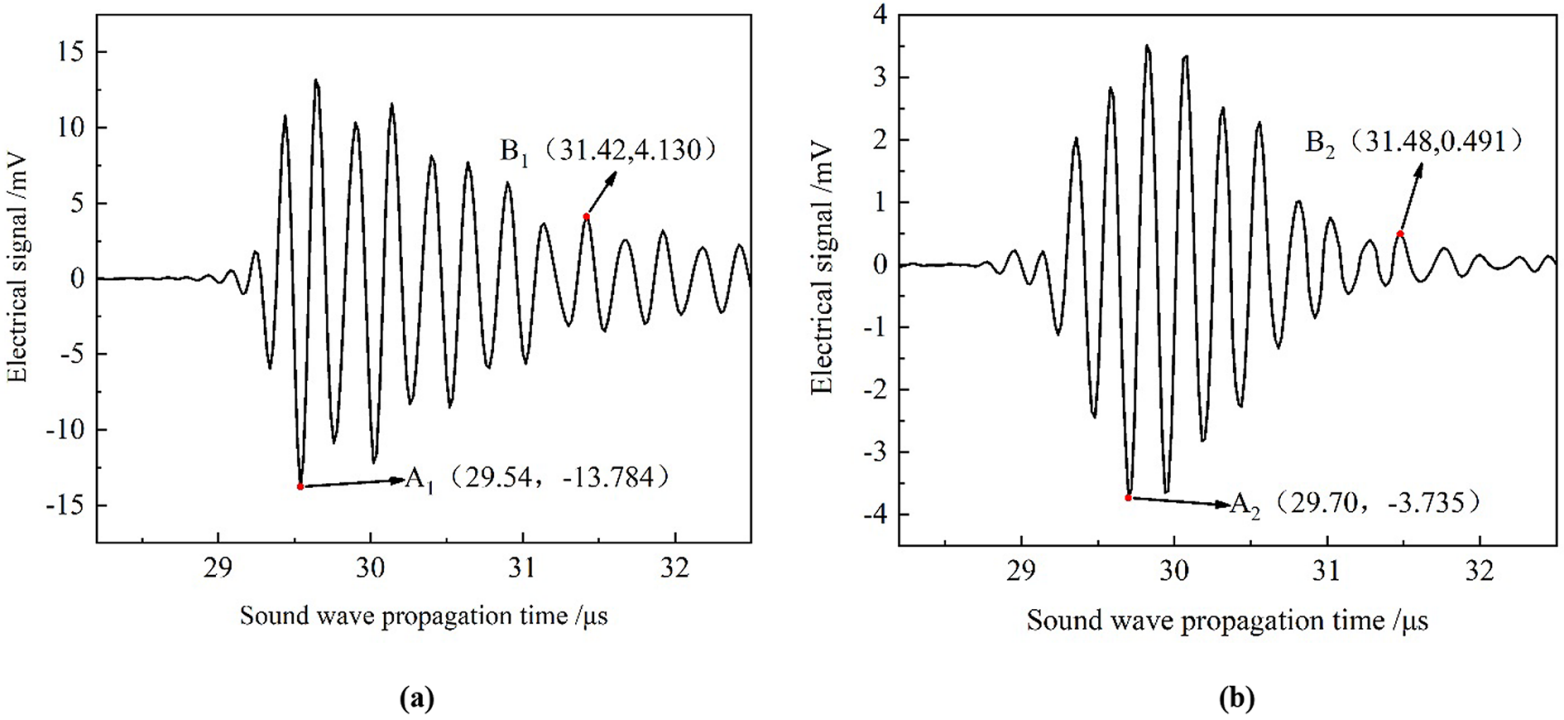
The simulated electrical signal diagram of two conversion methods when the circumferential deviation angle is 1.0°: (**a**) centre-symmetric conversion method; (**b**) optimized conversion method.

The relative orientation between the probe center and the defect center on the inner wall of the pipeline frequently changes during in-line inspection process of a pipeline. For instance, there exists a circumferential deviation angle between the probe and the defect center, as illustrated in Fig. 12. The angle α between lines CD and CE represents this circumferential deviation angle. To verify the accuracy of the optimized conversion method in cases where there is a circumferential deviation angle between the probe and the defective pipeline, the electrical signals obtained through the two methods were analyzed when the defect is the same as described in “Acoustic field distribution” Section, with a circumferential deviation angle of 1.0°, as shown in Fig. 13.

As shown in Fig. 13a, the maximum absolute value of the surface echo obtained through the centre-symmetric conversion method, referred to as point A1, is observed at (29.54 µs, − 13.784 V). The maximum absolute value of the primary bottom echo, designated as point B1, occurs at (31.42 µs, + 4.130 V). While in Fig. 13b, the maximum absolute value of the surface echo obtained through the optimized conversion method, denoted as point A2, is observed at (29.70 µs, − 3.735 V). The maximum absolute value of the primary bottom echo, represented as point B2, occurs at (31.48 µs, + 0.491 V). Following the same calculation approach as described in “Centre-symmetric conversion method” Section, the lift-off height from the probe to the inner wall of the pipeline and the wall thickness were determined for both conversion methods, as shown in Table 4. The optimized conversion method was found to result in an 5.90% improvement in the accuracy of the calculated wall thickness compared to the old method.

**Table 4:** Comparison of simulation results obtained by two methods and the measurements when the circumferential deviation angle is 1.0^o^.

Items	Lift-off height/mm	Relative error/%	Wall thickness/mm	Relative error/%
Measurement	21.40	/	5.08	/
Simulation by the old method	21.635	+ 1.10	5.64	+ 11.02
Simulation by the optimized method	21.753	+ 1.65	5.34	+ 5.12

## Conclusions

An optimized method for determining the initial excitation load in simulating the ultrasonic probe’s positive piezoelectric process. This method utilized the conversion of a 2D acoustic field into a 3D one, considering the focusing effect of the curved surface of pipelines on the ultrasonic signal generated by a piezoelectric ultrasonic probe. By employing both the new and old conversion method for initial excitation loads, the electrical signal characteristics were comparatively analyzed using COMSOL software under various conditions: when the probe is directly aligned with the pipeline, when the pipeline is defect-free and defective, and when there is a circumferential deviation angle between the probe and the defective pipeline. The model was validated against experimental data. The wall thickness of the pipeline was calculated based on the surface echo and the primary bottom echo. The results demonstrated that the optimized method for determining the excitation load could predict the wall thickness more accurately under all conditions. Consequently, it is essential to consider the focusing effect of the curved surface of pipelines on the ultrasonic signal when establishing a digital research and development platform for ultrasonic internal detectors of pipeline.

## Data Availability

The datasets generated and/or analyzed during the current study are available from the corresponding author on reasonable request.
